# Detection of the *Plasmodium falciparum* Kelch-13 gene P553L mutation in sporozoites isolated from mosquito salivary glands in South-Central Vietnam

**DOI:** 10.1186/s13071-017-2247-9

**Published:** 2017-06-24

**Authors:** Yoshimasa Maeno, Nguyen Tuyen Quang, Richard Culleton, Satoru Kawai, Gaku Masuda, Kaoru Hori, Shusuke Nakazawa, Ron P. Marchand

**Affiliations:** 1 Department of Virology and Parasitology, Fujita Health University School of Medicine, Toyoake, Aichi Japan; 2 Khanh Phu Malaria Research Unit, Medical Committee Netherlands-Viet Nam, Nha Trang, Khanh Hoa province Viet Nam; 3 Malaria Unit, Department of Pathology, Institute of Tropical Medicine, Nagasaki University, Nagasaki, Japan; 4 Laboratory of Tropical Medicine and Parasitology, Dokkyo Medical University, Mibu, Tochigi Japan; 5 Faculty of International Liberal Arts, Juntendo University, Tokyo, Japan; 6 Department of Protozoology, Institute of Tropical Medicine, Nagasaki University, Nagasaki, Japan

**Keywords:** *Plasmodium falciparum*, *Anopheles dirus*, Sporozoite, Artemisinin resistance, K13-propeller gene, Vietnam

## Abstract

**Background:**

*Plasmodium falciparum* has developed resistance against artemisinin in Southeast Asia. Mutations in the *P. falciparum* Kelch-13 (*Pfk13*) gene are associated with artemisinin resistance in vitro and in vivo. We investigated the prevalence of mutations in *PfK13* from sporozoite-stage parasites isolated from the salivary glands of *Anopheles dirus* mosquitoes.

**Methods:**

Mosquitoes were caught by human-landing catches at two locations within the Khanh Phu commune, South-Central Vietnam. Identification of *Anopheles* species was performed based on morphological features and nucleotide sequence analysis. Sporozoite-infected salivary glands were stored on filter paper and at 4–6 °C. A nested-PCR targeting the small subunit ribosomal RNA gene was used for *Plasmodium* species identification. *Pfk13* was amplified by nested PCR, and subjected to nucleotide sequencing.

**Results:**

Five of 33 *P. falciparum* sporozoite samples carried the P553L mutation at the *PfK13* locus*.* This mutation has been recorded previously in Vietnam, but not in Khanh Hoa province, were surveys of K13 polymorphism have not previously been carried out.

**Conclusion:**

These results demonstrate the utility of mosquito-stage malaria parasite samples for studies on the molecular epidemiology of drug resistance.

## Background

Artemisinin-based combination therapy (ACT) against *Plasmodium falciparum* is currently the most common and effective first-line therapy in most malaria-endemic countries. Recently, however, clinical cases of artemisinin resistance have been reported from various countries in Southeast Asia [1–6] and Africa [7].

Genome-wide analysis of artemisinin resistance in *P. falciparum* has demonstrated that mutations in the propeller domain of the gene encoding the Kelch 13 (K13) protein (*Pfk13*) are associated with delayed parasite clearance in vitro and in vivo [8, 9]. In Southeast Asia, a number of mutations in the *Pfk13* gene linked to artemisinin resistance have been identified, including C580Y, Y493H, R539T, I543T and others. In Vietnam, the C580Y, P574L, V568G, P553L, I543T and Y493H mutations have been recorded, all from the Southern part of the country on the border with Cambodia [10].

In this report, we characterized the polymorphism at the *Pfk13* locus using parasite DNA extracted from sporozoites isolated from the salivary glands of human-biting *Anopheles dirus* mosquitoes in South-central Vietnam.

## Methods

Collection of mosquitoes was carried out through human-baited landing catches in and around the forest near Nga Hai village in the south of Khanh Phu commune, Khanh Vinh district, Khanh Hoa province, Vietnam. Collection of mosquitoes was carried out as previously described [11, 12] from January 2008 to October 2012. Mosquito collectors provide informed consent and were regularly screened for malaria and treated with ACT if a malaria infection was detected. Identification of *Anopheles* species was carried out based on morphological characteristics [13] and through analysis of nucleotide sequences [12]. Female mosquitoes were dissected and examined by microscopy for sporozoites. Sporozoite-infected salivary glands were stored on filter paper kept in closed vials at 4–6 °C until analysis [13].

Genomic DNA (gDNA) was extracted from preserved filter paper with sporozoite-positive salivary glands [11, 14]. The 18S rRNA gene-based nested PCR was used for the detection of *P. falciparum* and other *Plasmodium* species [12, 15]. Amplification of the *Pfk13* gene was carried out by nested PCR as previously described [8], and the products sequenced with BigDye Terminator v3.1 Cycle Sequencing Premix Kit (ABI, Foster city, CA, USA). Sequencing products were run on an ABI/Hitachi 3130x1 Genetic Analyzer (ABI) and nucleotide sequences were analysed using Genetyx (Genetyx Corporation, Tokyo, Japan).

## Results and discussion

**Fig. 1:**
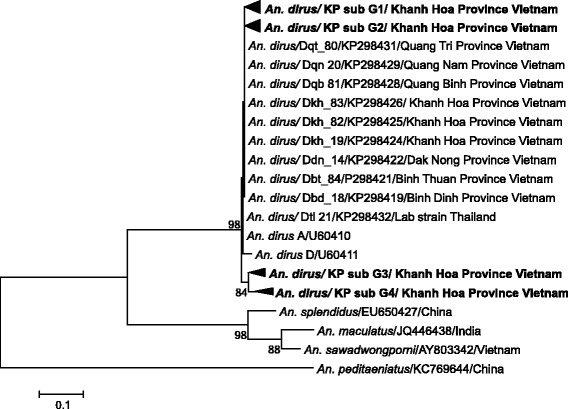
Molecular phylogenetic analysis of *Anopheles dirus* in Khanh Phu commune (KP subgroup 1, 2, 3 and 4) by maximum likelihood methodology. The evolutionary history was inferred by using the Maximum Likelihood method based on the Tamura-Nei model. The percentage of trees in which the associated taxa clustered together is shown next to the branches. Bootstrap values of < 80% are not shown. The scale-bar indicates the expected number of substitutions per nucleotide. Evolutionary analyses were conducted in MEGA6

A total of 11,464 female *An. dirus* (Figure 1) were captured; of these, 11,437 mosquitoes were dissected and the presence of sporozoites in salivary glands determined by microscopic examination. One hundred and sixty-eight (1.47%) showed *Plasmodium* sporozoites infection (Table 1). Of the sporozoites infected mosquitoes, 152 out of 168 (90.5%) were used for this study. By nested PCR analysis, 42 out of 152 stored sporozoite-positive mosquitoes (27.6%) were identified as harbouring *P. falciparum* (Table 2).

**Table 1:** Results of the collections and dissections of *Anopheles dirus* mosquitoes caught by human landing catch in the study area

Year	Period	No. caught	No. nights	Biting density^a^	No. dissected	No. sporozoites	%
2008	Jan to Dec	2120	724	2.9	2119	22	1.0
2009	Jan to Dec	2856	676	4.2	2848	32	1.1
2010	Jan to Dec	3218	674	4.8	3207	77	2.4
2011	Jan to Dec	1601	659	2.4	1594	21	1.3
2012	Jan to Oct	1669	563	3.0	1669	16	1.0
Total		11,464	3296	3.5	11,437	168	1.5

*Abbreviations*: Jan, January; Oct, October; Dec, December ^a^Average human-biting density (No. of caught/No. of caught person-night)

**Table 2:** Prevalence of *Plasmodium falciparum* and analysis of K13 propeller gene of sporozoites in *Anopheles dirus* mosquitoes

Year	No. examined samples	*P. falciparum*	K13 gene analyzed	Wild type (%)^a^	Mutation type (%)^a^
2008	7	2	2	2	0
2009	31	14	13	9	4
2010	77	23	15	14	1
2011	21	3	3	3	0
2012	16	0	0	0	0
Total	152	42	33	28 (84.8)	5 (15.2)

^a^Wild type (or mutation type) / K13 gene analysed

Nucleotide sequencing was performed on the nested PCR amplified portion of *Pfk13* (nucleotide positions 1279–2127) for 34 out of the 42 *P. falciparum* sporozoite-positive mosquitoes, of which 33 were successfully assayed (Table 2). Single nucleotide polymorphisms (SNPs) with respect to the *Pfk13* sequence of the 3D7 clone (PF3D7_1343700) were detected at low frequencies (Table 3). Synonymous mutations were not observed, and only a single non-synonymous mutation, P553L, was recorded. This mutation was present in 5 of the 33 *P. falciparum* sporozoite-positive samples analysed (15%). Of these samples, one was found to be a co-infection of wild type and mutant alleles, and the other four were single genotype mutant alleles. These five-mutant allele-positive samples were captured in March and April 2009 and 2010, and no mutant alleles were observed from mosquitoes captured at other times (Table 3). Samples containing mutant alleles were captured both in the forest and at the forest fringe. Three mutant samples were observed in *P. falciparum* single species infections, and two were found in *P. falciparum* infections co-infecting mosquito salivary glands with other malaria parasite species (Table 3).

**Table 3:** Mutant alleles of *Pfk13* in *Plasmodium falciparum* sporozoites from *Anopheles dirus* mosquitoes

ID	Year	Collected date	Parasite species	Nonsynymous			Synonymous
				Amino acid change and location	Genetic change	Captured site	Genetic change
70701	2009	3 March	Pf	P553L	CCG → CTG	Forest fringe	None
70752	2009	6 March	Pf	P553L	CCG → CTG	Forest fringe	None
70926	2009	15 March	Pf + Pk + Pinu	P553L	CCG → CTG	Forest fringe	None
71308	2009	3 April	Pf + Pcoat	P553L	CCG → CTG	In the forest	None
76280	2010	9 April	Pf + Pv + Pinu	P553L + Wild	CCG → CTG, None	In the forest	None

*Abbreviations*: *Pf, P. falciparum*; *Pv, P. vivax*; *Pk, P. knowlesi*; *Pinu, P. inui*; *Pcoat, P. coatneyi*

The prevalence of malaria in the study area has declined sharply since 1998 and was considered to be ‘residual forest malaria’ during the study period, while the proportion of *P. falciparum* among infections has remained substantial; densities of *An. dirus* typically peak in March and April [11]. The sample sizes of *P. falciparum* infections analyzed for K13 from 2008 to 2012 (Table 2) preclude analysis of prevalence trends through time.

There have been no previous reports of ART-resistance associated mutations in the *Pfk13* gene from this region of Vietnam. We observed only an azygous non-synonymous mutation in *Pfk13*, P553L, and this was present at low frequencies. This mutant allele has been identified previously, in samples collected from the Vietnam-Cambodia border, Southern China and Kenya [10, 16–19]. It is not currently known whether it is linked resistance to ART [8].

The Vietnamese national malaria treatment guidelines proscribe that patients are treated with 2.4 mg/kg of body weight dihydroartemisinin and 18 mg/kg piperaquine once a day for 3 days, and these guidelines were in effect in Khanh Hoa province during this study. The first record of the protracted parasite clearance after treatment with artesunate monotherapy or dihydroartemisinin-piperaruine was from the Bu Dang district of Binh Phuoc province in 2009 [20]. In that area, five types of mutant allele, including P553L, have been found *Pfk13* [10]. Our study demonstrates the presence of the *Pfk13* P553L SNP in 2009 and 2010 in Khanh Hoa province, some 200 km distant from Binh Phuoc. This result could suggest that the parasite populations of Southcentral Vietnam are contiguous over large distances, and that mutant allele gene flow is possible between relatively distant regions, although more evidence is required to support this hypothesis.

## Conclusions

In conclusion, this study demonstrates the presence of mono non-synonymous nucleotide mutations in the *Pfk13* gene in *P. falciparum* sporozoites isolated from the salivary glands of from *An. dirus*. The observed mutant allele, P553L, has previously been reported in a different region of Vietnam. No other mutant alleles, including those previously recorded in Vietnam were found in this study area.

Our results show that mosquito stage malaria parasites are a useful source of parasite DNA for drug-resistance associated molecular epidemiological studies.
